# Unraveling the interplay of DNA methylation and chromosome organization

**DOI:** 10.1042/BST20253094

**Published:** 2025-11-10

**Authors:** Yuhe Pei, Guoqiang Li

**Affiliations:** Biomedical Pioneering Innovation Center (BIOPIC), Beijing Advanced Innovation Center for Genomics (ICG), School of Life Sciences, Peking University, Beijing, 100871, China

**Keywords:** chromosome organization, DNA methylation, multi-omics method

## Abstract

Eukaryotic DNA has been covalently modified by DNA methylation and folded into a three-dimensional conformation in the nucleus. While the functions of DNA methylation and chromosome organization have been widely discussed, respectively, the interplay between DNA methylation and chromosome organization remains less clear and needs to be further explained. In this review, we first discuss the cross-talk between DNA methylation and chromosome conformation, highlighting the complexity and importance of DNA methylation on chromosome organization. We also summarize the current methodologies that capture DNA methylation and chromosome organization individually or simultaneously in bulk and single cells. These mechanistic and methodological advancements facilitate broad interest in unveiling the interplay between DNA methylation and chromosome organization.

## Introduction

Epigenetics refers to modifications that alter gene expression and phenotypes without changing the DNA sequence [1], which has shown crucial roles in regulating complex biological processes, including fertilization, early embryonic development, and aging [2–5]. In eukaryotic cells, these mechanisms involve multiple layers, including covalent modifications on DNA molecules like DNA methylation and chromosome modifications such as histone modifications and chromatin accessibilities [6–8]. Beyond these epigenetic modifications, chromosome conformation, which represents the folding of the chromosome fiber in the nucleus, also has critical roles in the above biological processes [9–11].

DNA methylation is a fundamental epigenetic modification that primarily involves adding a methyl group to the fifth carbon of the cytosine ring, resulting in the formation of 5-methylcytosine (5mC). In mammals, DNA methylation predominantly occurs at CpG dinucleotides, regions where a cytosine nucleotide is followed by a guanine nucleotide, which often gather with other CpG dinucleotides to form an area called CpG islands (CGIs) [12,13]. CGIs are typically hypomethylated and are located in ∼70% of gene promoters. Hypomethylation in promoters is associated with active transcription, whereas gene bodies and intergenic regions are generally hypermethylated [14–16]. The dynamics of DNA methylation are achieved through reversible enzymatic reactions that mediate the transition between methylation and demethylation. The establishment and maintenance of DNA methylation are mediated by a family of DNA methyltransferases (DNMT) enzymes, while the reversibility of DNA methylation is facilitated by the ten-eleven translocation (TET) enzyme-mediated active demethylation [17–19]. The TET enzymes can oxidize 5mC to 5-hydroxymethylcytosine, 5-formylcytosine, and 5-carboxylcytosine, some of which serve as intermediates, allowing cells to respond to developmental and environmental signals, fine-tuning gene expression and chromosome states [20].

Chromosome conformation is also a critical layer of epigenetic regulation where chromosomes form a hierarchical three-dimensional (3D) structure in the nucleus of eukaryotic cells. The hierarchical structure starts from the smallest unit, the nucleosome, to larger structures including chromosome loops, topologically associating domains (TADs), chromosome compartments, and the largest structure, chromosome territories [9,21,22]. Chromosome conformation is highly dynamic and regulated by both chromosome complexes and architectural proteins. For instance, ATP-dependent chromosome remodelers are responsible for the repositioning of nucleosomes to modulate chromosome conformation [23]. Chromosome architectural proteins, including CCCTC-binding factor (CTCF) and cohesin, mediate the loop-extrusion mechanism, in which the cohesion complex extrudes and is halted by CTCF binding on the chromosome, leading to the formation of chromosome loops and TAD boundaries [24,25]. The chromosome structural hierarchy and dynamic changes help ensure the precision and flexibility of gene regulation [26].

## Effects of DNA methylation on chromosome organization

As a covalent and prevalent epigenetic modification, DNA methylation has been shown to regulate chromosome organization in intricate ways [27,28]. We systematically categorize the effects of DNA methylation on chromosome organization into physical and intermediate effects, based on how methylation influences chromosome organization. The physical effects of DNA methylation on chromosome organization include altering DNA biophysical properties and modulating nucleosome dynamics and positioning. Intermediate effects occur through interactions with DNA-binding proteins like CTCF and transcription factors.

### Physical effects of DNA methylation on chromosome organization

**Figure 1:**
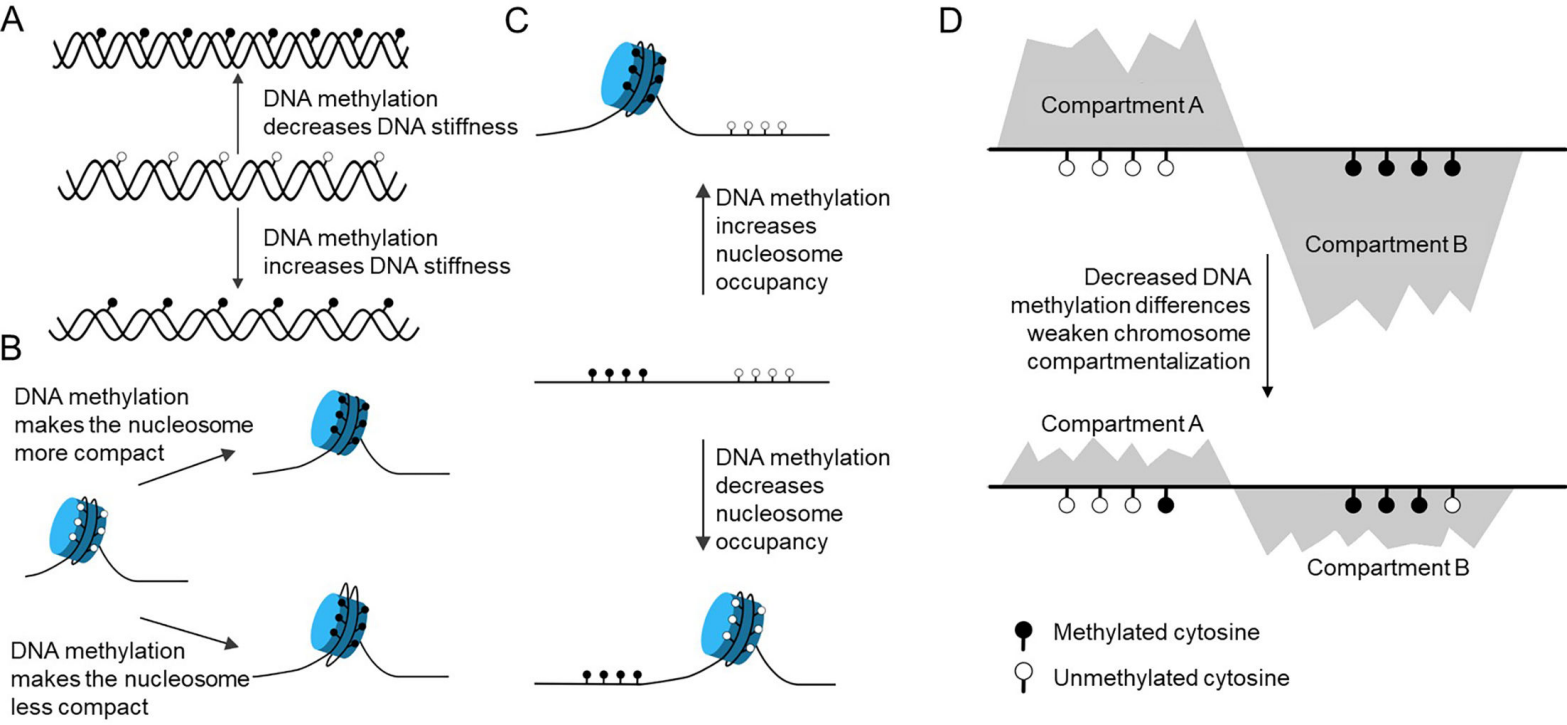
Physical effects of DNA methylation on chromosome organization. (**A**) The effect of DNA methylation on physical properties of DNA. DNA methylation may increase DNA stiffness or, on the contrary, decrease DNA stiffness. (**B**) The influence of DNA methylation on nucleosome dynamics. DNA methylation enhances the interactions of DNA and histone octamer to form more compact nucleosomes. Or DNA methylation makes nucleosomes less compact, and CpG methylation destabilizes nucleosomes. (**C**) The effect of DNA methylation on nucleosome positioning. Methylated CpG sites lead to an increased nucleosome occupancy, whereas unmethylated CpG sites tend to be nucleosome-depleted. Or DNA methylation accumulates in linker regions and exhibits an inverse correlation with nucleosome occupancy. (**D**) Changes in DNA methylation level weaken compartmentalization, corresponding to decreased methylation differences.

DNA methylation introduces a hydrophobic methyl group at the 5-carbon position of cytosine, which can alter DNA’s geometrical and chemical properties. However, due to the complexity of DNA sequence, histone contacts, and other factors, a consensus has not been reached on how methylation influences the physical properties of DNA. Several studies demonstrated that in a content-dependent manner, methylation reduced DNA flexibility and increased DNA stiffness through its influence on both base pairing and DNA backbone [29,30]. A calculation using molecular dynamics (MD) simulations, followed by validation of DNA circularization experiments, suggested that methylation at CpG sites modifies the curvature of the DNA molecule, making it less adaptable for bending [29]. However, many other studies draw the opposite conclusion that methylation can increase the ability of DNA to adopt bent conformations and reduce DNA stiffness [31,32]. With an improved magnetic tweezer-based method for precise force-extension measurements of DNA, researchers found that when focusing on CG-rich DNA, the methyl group led to an increase in local DNA flexibility [31] (Figure 1A). While the stiffness of CGIs can be additionally influenced by methylation, the correlation between DNA sequence, nucleosome occupancy, protein binding, and their methylation level has not been fully interpreted. Recent studies have demonstrated that the impact of DNA methylation on DNA bending is highly context-dependent. For instance, in free DNA, methylation can increase stiffness, reducing flexibility, as evidenced by MD simulations and experiments showing decreased dynamics of the DNA phosphate-sugar backbone upon methylation [33,34]. However, in the context of nucleosomal DNA, where DNA is wrapped around histone octamers, methylation may facilitate specific bending conformations, particularly under high curvature conditions [35]. Another research study even showed that methylation has contrasting effects on the stiffness of double strand DNA (dsDNA) depending on DNA curvature [36]. All these findings together demonstrate the importance of DNA methylation in DNA’s physical properties.

DNA flexibility affects nucleosome dynamics and mechanical stability [37]. With alteration of DNA properties, methylation can affect nucleosomes at various levels, including assembly, dynamics, and positioning [27,35,38], although the consequences are controversial. Some works suggest that methylation may enhance the interactions of DNA and histone octamer to form more compact nucleosomes. Two studies using single-molecule FRET and all-atom MD simulations suggest that methylation-induced DNA rigidity, bendedness, and narrowed minor grooves may facilitate extra contacts between DNA and the histone octamer, thereby suppressing DNA unwrapping [35,39]. These observations have also been validated in some genomics studies that analyze genome-wide nucleosome positioning and DNA methylation, which found that methylated CpG sites correspond to an increased nucleosome occupancy, whereas unmethylated CpG sites tend to be nucleosome-depleted [38,40]. However, some other studies obtained opposite results. Through experimental and computational methods, they find that DNA methylation makes nucleosomes less compact and CpG methylation destabilizes nucleosomes [30,41], in line with a study showing that DNA methylation typically accumulates in linker regions and exhibits an inverse correlation with nucleosome occupancy [28]. These results highlight methylation’s role in regulating chromatin accessibility, chromatin compaction, and function. The conflicting findings lean toward a complex, context-dependent relationship between DNA methylation and nucleosome occupancy. Specific genomic features, such as exons, promoters, or heterochromatin, can influence how methylation and nucleosomes interact [42]. Furthermore, differences in experimental approaches, including *in vitro*, *in vivo*, and *in silico* studies, and different model organisms used contribute to these discrepancies, drawing conflicting conclusions (Figure 1B–C).

The change of DNA methylation level can regulate chromosome organization, as global DNA hypermethylation leads to more condensed and isolated chromosomes. By inducing DNA methylation with the murine DNMT in budding yeast that lack intrinsic DNA methylation machinery, researchers found that DNA methylation could intrinsically modulate chromatin structure [28]. Chromosome conformation capture method Hi-C and 3D modeling revealed that the methylated yeast genome exhibits increased intra-chromosomal contacts, particularly near centromeres, and reduced inter-chromosomal interactions. This results in more rigid and condensed chromosomes, reduced overall DNA flexibility and interactions with other chromosomes, and enhanced heterochromatin state associated with gene silencing. Furthermore, the methylated yeast genome showed an elongated nucleus with denser internal chromosome packing, indicating a direct effect on nuclear morphology [28,43]. Similar results were found in plants [44]. Researchers focused on Arabidopsis found that highly methylated regions participate in fewer long-range chromatin contacts compared with regions with low methylation levels [45]. DNA methylation-defective mutants of Arabidopsis showed chromosome organization disturbance, including losses of interaction in pericentromeric regions and gains in interaction among interactive heterochromatin islands [46].

DNA methylation also affects chromosome compartmentalization. The chromosomes are generally compartmentalized into active and inactive compartments corresponding to compartment A and compartment B, respectively, and the switch of compartments often contributes to the activation or repression of specific genes [47,48]. DNA methylation is highly correlated with chromosome compartmentalization, as compartment A shows low methylation levels at CpG sites and compartment B corresponds to high DNA methylation levels [49,50]. Beyond methylation level, the CpG density distribution is also strongly associated with chromosome organization, with CGI-enriched domains corresponding to compartment A and CGI-depleted domains corresponding to compartment B [51,52]. Notably, the DNA methylation difference between CGI-rich and CGI-poor domains correlates very well with the strength of compartmentalization, suggesting that the methylation difference between CGI-rich and CGI-poor domains regulates compartmentalization [52,53]. In our previous work, we found that TET triple knock-out (TET-TKO) narrowed the methylation differences between CGI-rich and CGI-poor domains by inducing CGI hypermethylation, which corresponds to weakened chromosome compartmentalization (Figure 1D) [54]. In line with this observation, global loss of DNA methylation in DNMT double knock-out (DNMT-DKO) cells and 5-Azacitidine-treated cells, which removed methylation in both CGI-rich and CGI-poor domains, also showed a significant decrease in compartmentalization strength [55]. These results suggest a direct regulatory effect of DNA methylation on chromosome compartmentalization.

### Intermediate effects of DNA methylation on chromosome organization

**Figure 2:**
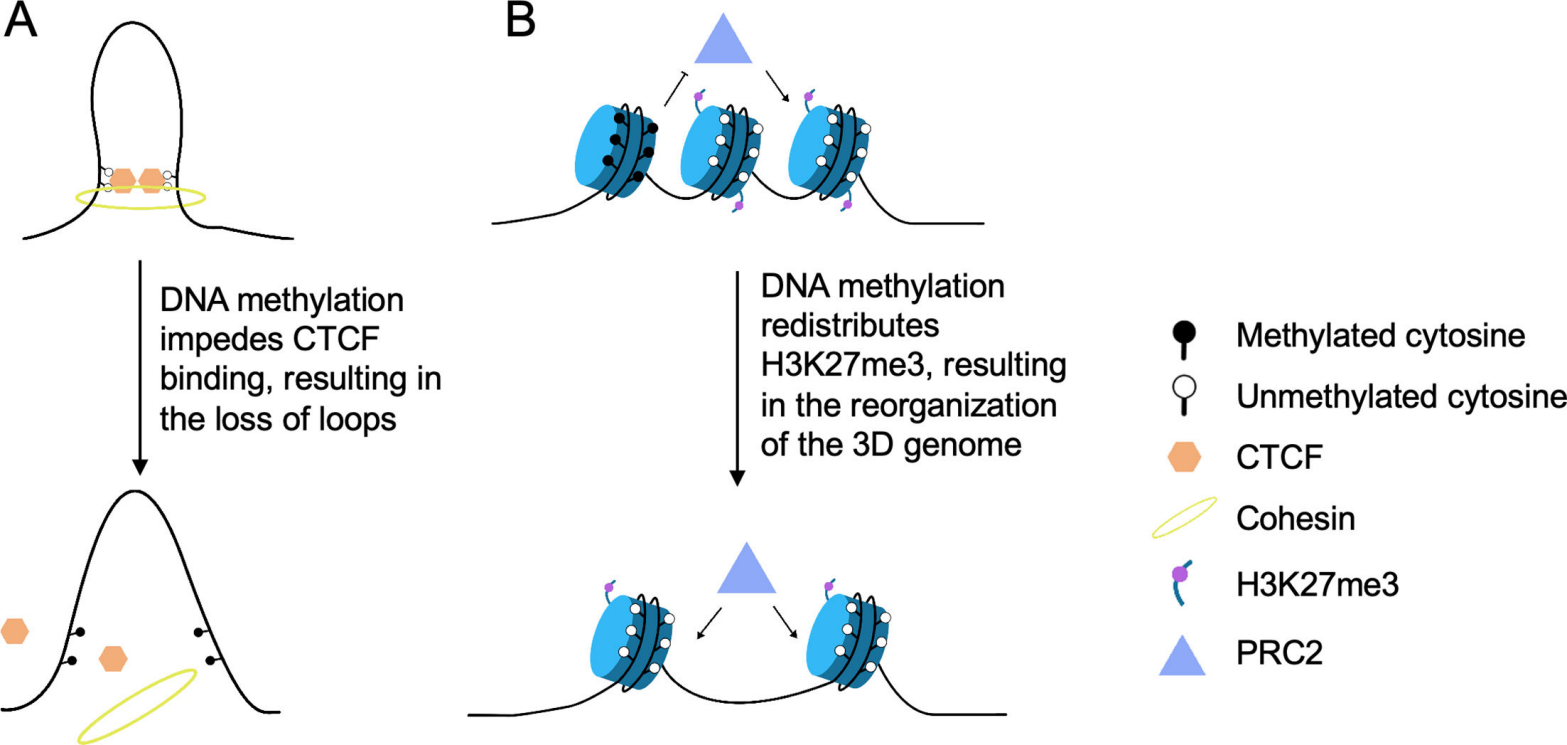
Intermediate effects of DNA methylation on chromosome organization. (**A**) Hypermethylation of CTCF-binding peaks impeded CTCF binding, resulting in the loss of long-range chromosome loops mediated by loop extrusion. (**B**) DNA hypomethylation leads to H3K27me3 redistribution and a reorganization of both local and long-range Polycomb-mediated chromosome contacts.

DNA methylation can also regulate chromosome organization by influencing DNA–protein interactions. The CTCF is one of the key architectural proteins in mediating chromosome loop extrusion to form TAD boundaries and chromosome loops for gene regulation [51,52]. CTCF binding on the chromosome is sensitive to DNA methylation, with the CTCF core motif containing cytosine residues prone to methylation [53]. One typical example showing the effects of DNA methylation on chromosome looping via modulating CTCF binding is the classical *Igf2*-*H19* imprinting domain [56,57]. The imprinting control region (ICR) is located between *Igf2* and *H19*, which contains a CTCF-binding motif. The intrachromosomal looping mediated by CTCF is crucial for regulating the entire maternal *Igf2*/*H19* imprinted region [51,58,59]. On the paternal allele, methylated ICR prevents CTCF binding and insulation, which allows the upstream enhancers to activate the *Igf2* gene expression but not *H19*. While on the maternal allele, the unmethylated ICR acts as an insulator by recruiting CTCF, which blocks communication between the *Igf2* promoter and enhancers. A similar effect is also observed by epigenome editing to add methylation at specific loci of the CTCF motif in human 293 T cell lines, which resulted in reduced CTCF binding and loss of looping mediated by enhancer–promoter interactions [60]. To assess the effects of DNA methylation on chromosome organization, our previous work employed mouse embryonic stem cells with methylation dioxygenase TET-TKO and a multi-omics strategy that captures DNA methylation and high-order chromosome interactions simultaneously [54]. We found that compared with wildtype (WT) cells, TET-TKO induced hypermethylation of CTCF-binding peaks at both TAD boundaries and chromosome loop anchors, which impeded CTCF binding and resulted in weakened TAD structure and long-range chromosome loops (Figure 2A) [61].

Beyond architectural proteins like CTCF, DNA methylation can also regulate chromosome conformation through other transcription factors and histone modification [62–64]. In a recent study, embryonic stem cells transitioning from serum to two small-molecule inhibitor culturing condition exhibited DNA hypomethylation, leading to H3K27me3 redistribution [65]. This redistribution was linked to a global change in the 3D genome organization, more specifically, a loss of both local and long-range Polycomb-mediated chromosome contacts. By preventing DNA hypomethylation during the cell transition, the study shows that it is possible to restore the H3K27me3 distribution and Polycomb-mediated 3D genome organization. A similar effect was also observed in hematopoietic stem cell, in which large DNA methylation nadirs or canyons can form megabase-long loops, connecting loci with repressive H3K27me3 marks made by the Polycomb complex such as polycomb repressive complex 2 (PRC2)^61^. These findings demonstrate that DNA methylation plays a significant role in shaping chromosome structure via directing Polycomb distribution (Figure 2B).

Recent advances in epigenome editing technologies, such as CRISPR/dCas9-based systems, have provided novel approaches to manipulate DNA methylation and demethylation in targeted regions, enabling researchers to determine relationships between DNA methylation and chromosome structure at specific genomic loci [66]. In a pivotal study, targeted DNA methylation editing was performed using inactive Cas9 (dCas9) fused to DNMT or demethylases in the mammalian genome. They found that targeted methylation of specific CTCF anchor sites by dCas9-Dnmt3a blocked CTCF binding and thus interfered with chromatin structure [67]. Furthermore, some relevant research suggested that the performance of targeting the DNA methylation editing system may vary due to the 3D structure of chromatin [68,69]. These advances in epigenome editing not only facilitate *in vivo* studies to make precise changes to DNA methylation and other epigenetic marks without altering the DNA sequence but also enhance our understanding of the relationship between DNA methylation and chromatin structure.

### Reciprocal effects of chromosome organization on DNA methylation

The relationship between DNA methylation and chromosome organization is bidirectional. The chromosome organization can also affect the DNA methylation distribution, which is crucial for biological processes such as gene expression and regulation [70–72]. Owing to the processivity of methyltransferases and demethylases, DNA methylation status has been observed to co-ordinate with their neighbors in local regions, which can be identified as methylation haplotype blocks [73]. The spatial chromosome contacts also create a shared microenvironment where linearly separated but spatially proximal genomic regions can be simultaneously exposed to the same DNMTs or TET enzymes, resulting in co-ordinated methylation status. By co-capturing of DNA methylation and chromosome interaction in single-molecule with multi-omics method Methyl-HiC, we observed a notable concordance of the methylation status of CpG sites in spatially proximal loop anchors [74]. Our recent work further found that the DNA methylation concordance of loop anchors was higher in TET-TKO cells than in WT cells, showing that less DNA methylation dynamics correlated with enhanced DNA methylation concordance in spatially interacted reads [54]. However, it is still hard to clarify whether concordant methylation statuses are a result of loop formation or a prerequisite for loop formation. More precise techniques, such as locus-specific genome editing coupled with imaging and DNA methylation profiling, are needed to decipher the complexity of the DNA methylation status in spatially interacting regions.

## Single-cell sequencing to capture DNA methylation and chromosome organization

One of the most significant findings from the Human Genome Project is that most of the human genome consists of non-coding regions with many cis-regulatory elements, which are crucial for spatial-temporal gene expression in specialized cell types for multicellular organisms [75–77]. With the development of sequencing technologies, especially single-cell sequencing technologies [78], we have revealed extensive cellular heterogeneity within complex tissues and comprehensive epigenetic mechanisms in gene regulation, including DNA methylation and chromosome organization [79,80].

The chromosome organization sequencing technologies are primarily based on DNA proximity ligation to identify chromosome contacts [81,82]. In 2009, by enhancing the sequencing library efficiency through a biotin pull-down step, the chromosome interaction map of the entire mammalian genome was revealed by the Hi-C technology [47,83]. This principle was further applied to single cells by isolating individual nuclei and performing index PCR amplification [84]. Multiple single-cell chromosome organization sequencing methods were developed in the following years and revealed extensive dynamic and heterogeneous chromosome organization in different cells [84–94].

The DNA methylation sequencing methods rely on converting methylated or unmethylated cytosine to a distinct base group. Currently, there are two main strategies for cytosine conversion: the bisulfite conversion and the enzymatic conversion [95]. The bisulfite conversion method has been viewed as the gold standard for DNA methylation detection with precise methylation quantification, but it suffers from significant DNA degradation during the conversion [40,96]. Recently, enzymatic conversion methods such as EM-seq and TET-assisted pyridine borane sequencing have been developed to minimize DNA damage and accommodate low DNA input [97,98]. The first single-cell DNA methylation sequencing method is single-cell reduced-representation bisulfite sequencing, which covers more than one million CpG sites per cell and reveals a fine demethylation landscape of post-fertilization embryos in mice [99]. Later on, single-cell whole-genome DNA methylation profiling based on bisulfite conversion was developed to recover ∼20% of all CpG sites in the mouse genome [100]. To achieve high throughput for cell processing, single-cell methods based on combinatorial indexing or droplet platforms were also developed for DNA methylation profiling, which enable high-throughput analysis of thousands of cells [101–103].

**Figure 3:**
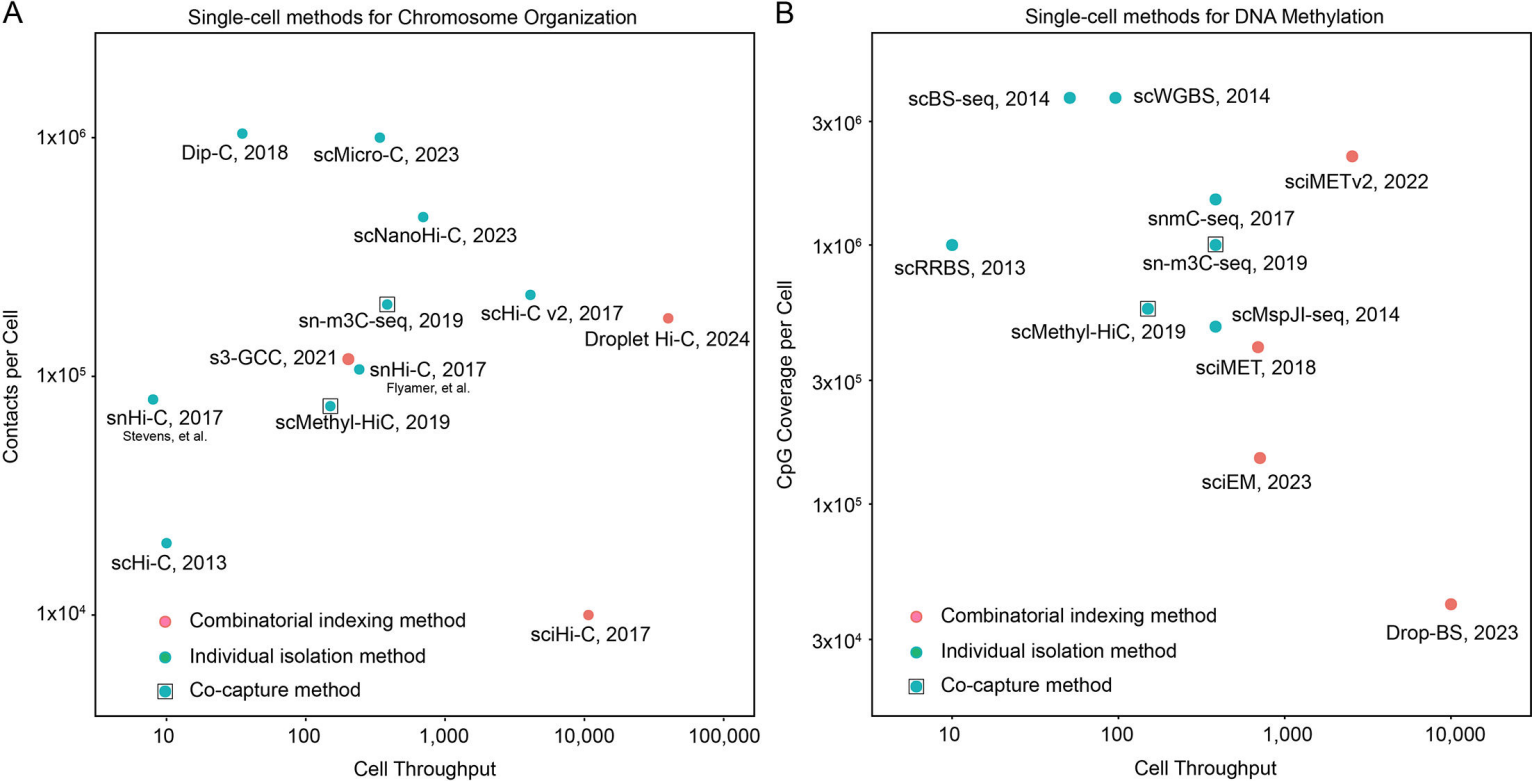
Comparison of single-cell chromosome conformation capture and DNA methylation methods. (**A**) Comparison of cell throughput and average contacts captured per cell for single-cell DNA chromosome conformation capture methods. (**B**) Comparison of cell throughput and CpG coverage per cell for some single-cell DNA methylation methods.

There is often a trade-off between cell processing throughput and modality capture efficiency in each cell. Technically, the above single-cell methods can be generally divided into isolation-based and combinatorial-indexing-based approaches to achieve single-cell resolution. The isolation-based methods typically achieve high contact or cytosine capture efficiency but have limited cell processing ability. On the other hand, the combinatorial-indexing-based methods enable higher processing throughput at the cost of lower contact or cytosine capture efficiency. Here, we summarize the single-cell chromosome conformation capture [84–94] and single-cell DNA methylation [99,100,102–106] methods developed recently and compare their cell throughput and average modality captured in the cell (Figure 3). We could anticipate that these challenges in single-cell sequencing will be progressively addressed in the future with alternative strategies.

## Joint analysis of DNA methylation and chromosome organization in single cells

As single-cell methods advanced, single-cell multi-omics techniques have emerged to simultaneously capture two or more modalities from individual cells, providing a more comprehensive view of cellular heterogeneity and modalities integration. Recent methods that can simultaneously profile DNA methylation and chromosome architecture in the same single cells have provided ways to study the interplay of DNA methylation and chromosome architecture in single cells [74,107]. Briefly, these methods perform bisulfite conversion on proximal-ligated products from capture chromatin conformation (3C) or Hi-C DNA at the single-cell level to simultaneously capture DNA methylation and chromosome interactions. One key advantage of co-capturing is to use DNA methylation to identify cell types for cell-type-specific chromosome organization analysis. Using DNA methylation from scMethyl-HiC data, we could cluster mouse embryonic stem cells into three heterogeneous populations with distinguished DNA methylation and chromosome conformation features [74]. Meanwhile, snm3C-seq profiled DNA methylation and chromosome conformation of more than four thousand cells from the adult human prefrontal cortex. They found that chromosome conformation data provide lower clustering resolution compared with DNA methylation. With the help of DNA methylation, they demonstrated that cell-type-specific chromosome interactions correlate with differential DNA methylation signals, suggesting pervasive interactions between epigenetic processes regulating cell-type-specific gene expression [107].

The co-assays have been extensively applied to profile DNA methylation and chromosome organization across diverse cell types in mouse and human tissues, generating valuable and extensive data resources to elucidate the epigenetic mechanisms of development and disorders [108–111]. Using snm3C-seq, Heffel et al. captured 53,000 nuclei of the human hippocampus and prefrontal cortex from mid-gestation to adulthood, revealing the temporally decoupled dynamics of DNA methylation and chromosome organization in human brain development [110]. After fine cell classification, they found that a neural progenitor radial glia (RG) population defined by DNA methylation modality in gestational brains can be further discretely divided into a neurogenic population and a putative astrocyte progenitor population using chromosome conformation signatures. During RG differentiation into astrocytes, CG methylation dynamics occurred significantly later than chromosome contact shifts, with two types of RG populations exhibiting asynchrony in DNA methylation and chromosome organization. Furthermore, this study focused on schizophrenia-associated genetic variants across developmental stages and cell populations, and they found that ∼30% overlapped chromosome loops connected differentially methylated regions (DMRs). Enrichment of polygenic heritability for DMRs and loop-connected DMRs in schizophrenia indicates that the genetic risk of schizophrenia more strongly affects late-gestational neurons than neuronal progenitors in developing human brains. Recently, the snm3C-seq has also been applied to multiple human adult tissues and revealed globally correlated but cell-type-specific discrepancies between DNA methylation and chromosome organization, indicating that the role of distinct epigenomic features in maintaining cell identity may vary by lineage [112].

We summarize recent studies employing single-cell multi-omics techniques to elucidate the interplay between DNA methylation and chromosome organization, highlighting their key findings (Table 1). These works further proved the importance of joint profiling of DNA methylation and chromosome conformation in single cells. However, existing single-cell co-assays still face the challenge of low cell throughput and complicated experimental procedures, hindering the broad application of these methods in more conditions. With the development of single-cell methods, we anticipate the advent of new multi-omics technologies to achieve high throughput and provide valuable insights into the relationship between DNA methylation and chromosome organization.

**Table 1:** Summary of single-cell multi-omics studies to elucidate the interplay of DNA methylation and chromosome organization

Reference	Year	Tissue/Cell	Description
Li G. et al. [74]	2019	Mouse embryonic stem cell	Presents Methyl-HiC, reveals co-ordinated methylation between spatially close genomic segments
Lee D.-S. et al. [107]	2019	Adult human prefrontal cortex	Introduces sn-m3C-seq, reconstructs cell-type specific chromosome maps from 14 cortical cell types
Tian W. et al. [109]	2023	Adult human brain	Examines 517 k cells, identifies 188 brain cell types, reveals concordant methylation and chromosome changes
Liu H. et al. [108]	2023	Adult mouse brain	Generates 301,626 methylomes and 176,003 chromosome profiles of mouse cells, identifying 2.6M DMRs
Zemke N.R. et al. [111]	2023	Adult mouse brain	Compares methylation and chromosome across species, shows human-specific cis-elements, reflects 3D genome evolution.
Heffel M.G. et al. [110]	2024	Developing human frontal cortex and hippocampus	Investigates temporal dynamics, finds DNA methylation remodeling separated from chromosome conformation changes
Zhou J. et al. [112]	2025	Human body	Profiles 86,689 nuclei across 16 human tissues, reveals extensive changes in CG and non-CG methylation and characterizes 3D chromosome organization

## Conclusion

DNA methylation influences chromosome organization through physical and intermediate mechanisms. Physically, methylation alters DNA properties and regulates nucleosome dynamics and positioning. It can also contribute to chromosome compartmentalization and condensation. DNA methylation can indirectly affect chromosome interactions by modulating loop extrusion via impeding CTCF binding on the chromosome or by modulating repressive histone modifications and Polycomb redistribution. These effects collectively regulate gene expression and maintain cellular identity, which can be further revealed by advanced single-cell chromosome conformation capture and DNA methylation methods in both development and disease.

**Perspectives**

- DNA methylation and chromosome organization are two important epigenetic mechanisms that regulate cellular functions. While each has been extensively studied, the cross-talk between DNA methylation and chromosome organization is controversial and has not been fully conveyed.
- DNA methylation can modulate chromosome organization by directly changing the physical properties of DNA and nucleosome assembly or indirectly affecting the recruitment of chromosome-binding proteins such as CCCTC-binding factor and transcription factors. Reciprocal influence of chromosome interactions can also affect methylation patterning.
- The emergence of single-cell and single-molecular multi-omics methods to simultaneously capture DNA methylation and chromosome interactions will provide great tools to investigate the interplay of DNA methylation and chromosome organization in the future.

## Open Access

This article has been published open access under our Subscribe to Open programme, made possible through the support of our subscribing institutions, learn more here: https://portlandpress.com/pages/open_access_options_and_prices#conditional

## Abbreviations

3C: capture chromatin conformation
CGIs: CpG islands
CTCF: CCCTC-binding factor
3D: three-dimensional
DMRs: differentially methylated regions
DNMT: DNA methyltransferases
DNMT-DKO: DNMT double knock-out
ICR: imprinting control region
MD: molecular dynamics
PRC2: polycomb repressive complex 2
RG: radial glia
TADs: topologically associating domains
TET: ten-eleven translocation
TET-TKO: TET triple knock-out
WT: wildtype
dCas9: inactive Cas9
dsDNA: double strand DNA
5mC: 5-methylcytosine
